# Patients with previous anterior cruciate ligament reconstruction achieve equally good outcomes after total knee arthroplasty: A Swedish registry‐based study of 7755 patients

**DOI:** 10.1002/ksa.70240

**Published:** 2025-12-23

**Authors:** Ioannis Syrikas, Ioannis Karikis, Neel Desai, Georgios Tsikandylakis

**Affiliations:** ^1^ Department of Orthopaedics Region Västra Götaland, NU‐Hospital Group Trollhättan/Uddevalla Sweden; ^2^ Department of Orthopaedics Institute of Clinical Sciences, Sahlgrenska Academy University of Gothenburg Sweden; ^3^ Department of Research and Development Region Västra Götaland, NU‐Hospital Group Trollhättan Sweden; ^4^ Department of Orthopaedics Region Västra Götaland, Sahlgrenska University Hospital Gothenburg Sweden

**Keywords:** anterior cruciate ligament reconstruction, osteoarthritis, patient‐reported outcome measures, posttraumatic osteoarthritis, total knee arthroplasty

## Abstract

**Purpose:**

Patients who have undergone anterior cruciate ligament reconstruction (ACLR) may later require total knee arthroplasty (TKA) due to osteoarthritis (OA). The impact of previous ACLR on patient‐reported outcomes (PROs) after TKA remains uncertain. This study compared PROs 1 year after TKA between patients with knee OA with a history of ACLR (group of patients who underwent total knee arthroplasty after anterior cruciate ligament reconstruction [ACLROA]) and those without prior major surgery on the operated knee (controls).

**Methods:**

The Swedish Arthroplasty and Knee Ligament Registers were linked. ACLROA cases were matched 1:2 to controls by age, sex, BMI, ASA score, implant type and surgery time period. Preoperative and 1‐year postoperative patient‐reported outcome measures (PROMs) included five‐point Likert scales for satisfaction and pain, knee injury and Osteoarthritis Outcome Score, 12‐item short form (KOOS‐12), and EuroQol 5‐dimensions, 3‐level version (EQ‐5D‐3L).

**Results:**

Cohort sizes varied for the analysis of PROMs: satisfaction was compared between 998 ACLROA patients and 1996 controls, pain between 441 and 882, KOOS‐12 between 595 and 1190, and EQ‐5D‐3L between 551 and 1102, respectively. At 1 year postoperatively, satisfaction scores differed significantly (*p* = 0.016), with a higher proportion of ACLROA patients reporting being ‘very satisfied’ (53% vs. 49%), although pain scores did not differ (*p* = 0.263). The mean KOOS‐12 total score was 59 ± 16 points for ACLROA patients and 58 ± 16 for the controls (*p* = 0.22). The mean EQ‐5D‐3L index was 0.89 ± 0.11 for ACLROA patients and 0.88 ± 0.11 for the controls (*p* = 0.019).

**Conclusions:**

Prior ACLR did not compromise early PROs after subsequent TKA. Satisfaction, pain relief, knee function and HRQoL were comparable between groups. Although some differences reached statistical significance, they did not exceed established thresholds for clinical relevance. Prior ACLR should therefore not be considered a negative prognostic factor regarding PROs during preoperative counselling for TKA.

**Level of Evidence:**

Level III.

**Abbreviations**
ACL: anterior cruciate ligament
ACLR: anterior cruciate ligament reconstruction
ACLROA: group of patients who underwent total knee arthroplasty after anterior cruciate ligament reconstruction
ASA score: American Society of Anesthesiologists physical status classification system
BMI: body mass index
CCK: constrained condylar knee
CI: confidence intervals
Controls: control group of patients with osteoarthritis undergoing total knee arthroplasty with no prior major surgery to the operated knee, such as osteotomy, osteosynthesis or anterior cruciate ligament reconstruction
CR: cruciate retaining
EQ‐5D‐3L: EuroQol 5‐dimensions, 3‐level version
EQ‐5D‐5L: EuroQol 5‐dimensions, 5‐level version
EQ‐VAS: EuroQol visual analogue scale
HRQoL: health‐related quality of life
KOOS‐12: knee injury and Osteoarthritis Outcome Score, 12‐item short form
KSS: Knee Society Score
MIC: minimal important change
OA: osteoarthritis
PN: personal identification number
PROMs/PROs: patient‐reported outcome measures/patient‐reported outcomes
PS: posterior stabilised
SAR: Swedish Arthroplasty Register
SD: ptandard deviation
SKLR: Swedish Knee Ligament Register
SPSS: Statistical Package for the Social Sciences
TKA: total knee arthroplasty
VAS: visual analogue scale

## INTRODUCTION

Injury to the anterior cruciate ligament (ACL) is a common cause of knee instability, predisposing to subsequent injuries, such as chondral damage or meniscal tears, functional loss, and early onset of osteoarthritis (OA) [8]. ACL reconstruction (ACLR) is the treatment of choice in patients with ACL ruptures who are candidates for operative management. While ACLR yields favourable outcomes in many patients [2], failure to fully restore normal knee kinematics may lead to subsequent degenerative changes in the knee joint, increasing the risk of OA [13, 20]. Nearly one‐third of patients develop radiographic or symptomatic OA after ACLR [9, 27], and ACL‐injured patients have been shown to undergo total knee arthroplasty (TKA) at a younger age, often with higher intraoperative complexity and complication rates [1].

Many patients with a history of ACLR are cautioned about potentially poorer TKA outcomes, although limited evidence from small, single‐centre studies has failed to support this praxis [7]. There is therefore a need for larger‐scale, register‐based studies to provide stronger evidence on whether patients with previous ACLR should expect inferior results after a TKA compared with the general knee OA population.

The present study aimed to explore whether a history of ACLR is a negative prognostic factor by comparing patient‐reported outcome (PROs) after TKA between patients with previous ACLR and those without a history of ACLR in a larger dataset, derived from the Swedish Arthroplasty Register (SAR) [25] and the Swedish Knee Ligament Register (SKLR) [26]. In light of altered knee kinematics, younger age at the time of TKA, and potentially higher patient expectations, it was hypothesised that prior ACLR could be associated with inferior PROs 1 year after TKA.

## METHODS

### Study design

This study utilised an observational, register‐based matched cohort design by integrating data from the SAR and the SKLR. Through this linkage, a more complete dataset was created regarding the history of ACLR.

### Setting

In 2021, the SAR was formed by merging the Swedish Knee Arthroplasty Register (SKAR) and the Swedish Hip Arthroplasty Register. Reporting for healthcare units achieves >97% coverage [25], and data are available for knee replacement procedures performed since 1975. The SKLR, established in 2005, monitors outcomes of knee ligament injuries and treatments, with >90% coverage of all ACLRs in Sweden and a unit‐level inclusion rate of 92.9% [11]. Data from the SAR and SKLR were extracted by the Centre of Registers in Västra Götaland County [6], including each patient′s unique Swedish personal identification number (PNs), and pseudonymized before release. Although the SAR records previous ACLR as a binary variable, its accuracy may be limited by underreporting, for example, of an ACLR that occurred long before a TKA. To improve data reliability, the SAR records were linked to the SKLR data from 2005 to 2018 using the pseudonymized PNs.

### Participants

**Figure 1:**
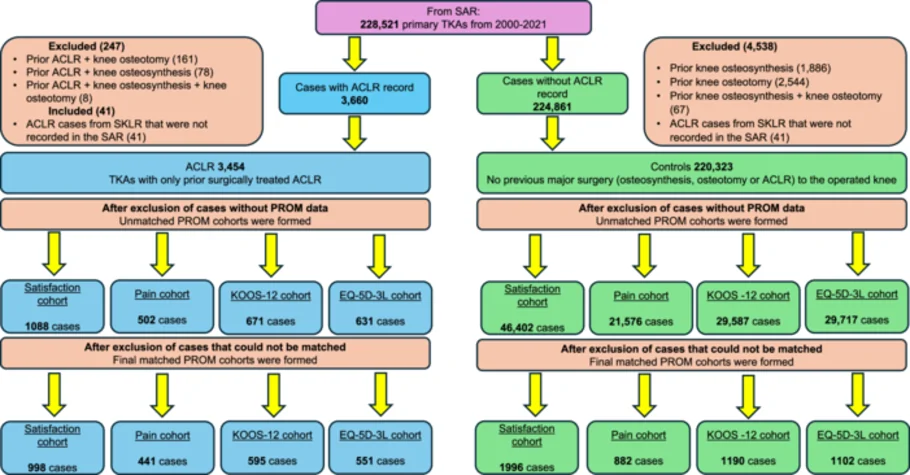
Flowchart of patients included in the study (ACLROA vs. controls). ACLROA, group of patients who underwent TKA after anterior cruciate ligament reconstruction; Controls, control group of patients with osteoarthritis undergoing TKA with no prior major surgery to the operated knee, such as osteotomy, osteosynthesis or anterior cruciate ligament reconstruction. ACLR, anterior cruciate ligament reconstruction; EQ‐5D‐3L, the 3‐level version of EQ‐5D—a standardised measure of health‐related quality of life developed by the EuroQol Group; KOOS‐12, a 12‐item short form of the Knee injury and Osteoarthritis Outcome Score; PROM, patient‐reported outcome measures; SAR, Swedish Arthroplasty Register; TKA, total knee arthroplasty.

The study population was divided into two groups. The first group, referred to as ACLROA, included adult patients from the SAR who underwent TKA between 2000 and 2021 and had a documented history of ACLR, as well as 41 patients who had undergone TKA but had an unregistered ACLR in the ipsilateral knee in the SAR. In total, the ACLROA group included 3454 patients after excluding those who, apart from ACLR, also had a history of other major knee surgery, such as surgical treatment of fracture or osteotomies around the same knee, meniscal surgery, knee arthroscopy or any other knee surgery recorded as ‘other’ in the SAR or SKLR. Patients with any previous ligament reconstruction recorded in the SKLR were also excluded. Only patients with complete pre and postoperative PROMs data—apart from patient satisfaction, which is reported only postoperatively—were included in the analysis, resulting in four ACLROA cohorts based on each PROM, with some minimal overlap. Thus, the ACLROA group comprised four cohorts based on each PROM, with 1088 cases in the satisfaction cohort, 502 in the pain cohort, 671 in the KOOS‐12 [12] cohort, and 631 in the EuroQol 5‐dimensions, 3‐level version (EQ‐5D‐3L) [21] cohort before matching (Figure 1). The four PROM cohorts included different patients from different reporting clinics because there was no uniform nationwide PROM program until the merger of the Swedish Hip and Knee Registries in 2021.

The second group served as a control group and consisted of patients with OA from the SAR who underwent TKA without any history of major surgery or any other knee surgery on the operated knee reported in SLR or SKLR. Major surgery was defined as ACLR, osteotomy or osteosynthesis around the knee. In total, 220,323 control cases were available before PROM‐based exclusions and matching. This group included 46,402 in the satisfaction cohort, 21,576 cases in the pain cohort, 29,587 in the KOOS‐12 cohort, and 29,717 in the EQ‐5D‐3L cohort before matching (Figure 1).

### Study variables

The data retrieved from the SAR included details such as age and body mass index (BMI) at the time of the index TKA, sex, operated side, and the surgery date. Additionally, information was collected on the indications for surgery, the type of knee implant used (cruciate retaining [CR]), posterior stabilised [PS], constrained condylar knee [CCK] or hinged), previous surgeries on the knee joint, and PROMs data, which were recorded preoperatively and at 1 year postoperatively. PROMs included five‐point Likert scales for satisfaction and pain (1 = worst, 5 = best), knee function measured with the KOOS‐12 (0–100, higher scores indicate better function), and health‐related quality of life (HRQoL) measured with the EQ‐5D‐3L index (0–1; 0 = health state equivalent to death and 1 = full health) together with the EQ‐VAS (0–100; 0 = worst health and 100 = best self‐reported health). As noted, PROMs reporting became standardised nationally after the SAR was created in 2021. Knee pain and patient satisfaction, previously measured with visual analogue scales (VAS), were thereafter measured with five‐point Likert scales. Data were only available for the EQ‐5D‐3L until 2020, after which the five‐level version (EuroQol 5‐Dimensions, 5‐Level version [EQ‐5D‐5L]) was used. The collection of the remaining PROMs extended through 2021.

The 1‐year postoperative time point was selected as the primary endpoint because it represents the standardised follow‐up interval in the SAR and corresponds to the phase in which functional recovery and symptom improvement after TKA are considered to have achieved their maximal recovery with minimal further change thereafter [23].

### Matching

To create comparable groups, ACLROA patients were matched to controls based on sex, age, BMI, ASA score, type of TKA implant (CR, PS, CCK or hinged), and according to the time period of the TKA procedure (≤2015 or >2016). The 2015 cut‐off reflects the year when PROM completeness and reporting stability in the SKAR improved markedly [25] and reached internationally recommended completeness levels [22].

Using the aforementioned variables, 1:2 case‐control matching was conducted. Exact matching was applied for sex, ASA score, implant type and time period of the TKA procedure. For age and BMI, matches were accepted within a permissible range of ±2 years and ±1 unit, respectively. These criteria resulted in a caliper width of 0.23 for both age and BMI, values supported by the literature as providing optimal treatment effect estimation, thereby ensuring closely matched pairs and minimising bias due to potential confounding by age and BMI [18]. Because reporting of PROMs varied across clinics, the matching procedure was performed separately for each PROM cohort (satisfaction, pain, KOOS‐12 and EQ‐5D‐3L) to ensure that each analysis was based on complete datasets. Following the matching process, the ACLROA and control groups were found to be well‐matched in terms of the relevant demographic and surgery‐related variables (Table 1). Matching quality was assessed descriptively by comparing baseline variables between groups before (Supporting Information: Table S1) and after matching (Table 1); close similarity of means, dispersions and proportions was considered indicative of adequate covariate balance.

**Table 1:** Demographic characteristics of matched PROMs cohorts.

Factor	Satisfaction Likert scale		Pain Likert scale		KOOS‐12		EQ‐5D‐3L	
	ACLROA	Controls	ACLROA	Controls	ACLROA	Controls	ACLROA	Controls
Number of cases	998	1996	441	882	595	1190	551	1102
Age mean (SD)	60.9 (7.9)	61.9 (7.7)	61.1 (7.4)	61.9 (7.3)	60.6 (7.7)	61.5 (7.6)	60.8 (7.8)	61.8 (7.7)
BMI mean (SD)	28.1 (3.7)	28.1 (3.7)	28 (3.5)	28 (3.5)	28.2 (3.6)	28.3 (3.6)	28.1 (3.6)	28.2 (3.6)
Sex *n* (%)								
Male	668 (67)	1336 (67)	284 (64)	568 (64)	391 (66)	782 (66)	363 (66)	726 (66)
Female	330 (33)	660 (33)	157 (36)	314 (36)	204 (34)	408 (34)	188 (34)	376 (34)
Side *n* (%)								
Right	516 (52)	1034 (52)	357 (81)	817 (93)	304 (51)	615 (52)	278 (51)	604 (55)
Left	482 (48)	962 (48)	84 (19)	65 (7.4)	291 (49)	575 (48)	273 (50)	498 (45)
ASA score *n* (%)								
1	328 (33)	656 (33)	136 (31)	272 (31)	198 (33)	396 (33)	184 (33)	368 (33)
2	594 (60)	1188 (60)	278 (63)	556 (63)	359 (60)	718 (60)	331 (60)	662 (60)
3	76 (7.6)	152 (7.6)	27 (6.1)	54 (6.1)	38 (6.4)	76 (6.4)	36 (6.5)	72 (6.5)
4	0 (0)	0 (0)	0 (0)	0 (0)	0 (0)	0 (0)	0 (0)	0 (0)
5	0 (0)	0 (0)	0 (0)	0 (0)	0 (0)	0 (0)	0 (0)	0 (0)
Type of articulation *n* (%)								
CR	947 (95)	1894 (95)	424 (96)	848 (96)	581 (98)	1162 (98)	535 (97)	1070 (97)
PS	47 (4.7)	94 (4.7)	13 (2.9)	26 (2.9)	12 (2)	24 (2)	14 (2.5)	28 (2.5)
CCK	4 (0.4)	8 (0.4)	4 (0.9)	8 (0.9)	2 (0.3)	4 (0.3)	2 (0.4)	4 (0.4)
Hinged	0 (0)	0 (0)	0 (0)	0 (0)	0 (0)	0 (0)	0 (0)	0 (0)
Year of TKA *n* (%)								
2000–2015	171 (17)	342 (17)	76 (17)	152 (17)	138 (23)	276 (23)	155 (28)	310 (28)
2016–2021	827 (83)	1654 (83)	365 (83)	730 (83)	457 (77)	914 (77)	396 (72)	792 (72)

*Note*: Mean (standard deviation), absolute (relative frequencies). *p*‐Values are not reported for matched comparisons, as significance testing is not recommended for assessing balance due to its dependency on sample size. Balance was evaluated descriptively by comparing means and proportions between groups [3, 24].

Abbreviations: ACLROA, group of patients treated with total knee arthroplasty following prior anterior cruciate ligament reconstruction; ASA score, American Society of Anaesthesiologists Classification; BMI, body mass index; CCK, constrained condylar knee; Controls, control group of patients with osteoarthritis undergoing total knee arthroplasty with no prior major surgery to the operated knee, such as osteotomy, osteosynthesis or anterior cruciate ligament reconstruction; CR, cruciate retaining; EQ‐5D‐3L, the 3‐level version of EQ‐5D is a standardised measure of health‐related quality of life developed by the EuroQol Group; KOOS‐12, a 12‐item short form of the Knee injury and Osteoarthritis Outcome Score; PROMs, patient‐reported outcome measures; PS, posterior stabilised; TKA, total knee arthroplasty.

Matching was selected over multivariable regression adjustment to achieve direct comparability between the ACLROA and control groups, limit the analyses to comparable cases, and minimise model dependence and extrapolation beyond the region of covariate overlap [3, 24]. A 1:2 ratio was chosen to increase statistical sensitivity and maintain balance between groups. Adding more than two controls per case would provide minimal further gain in precision [4].

### Statistical analysis

Continuous data were presented as means with their standard deviation (SD), whereas categorical data were presented as counts and percentages. All statistical analyses were conducted with IBM SPSS Statistics, version 29.0. Analyses were based solely on complete cases; no methods of imputation were applied. Patients lacking information required for matching or outcome assessment were removed during cohort assembly (Figure 1).

Pearson's chi‐square test was used to assess the statistical significance of the group differences in patient satisfaction and pain (five‐point Likert scales). For the continuous scores (KOOS‐12 and EQ‐5D‐3L), the Kolmogorov–Smirnov test indicated departures from normality; consequently, group differences were examined with the nonparametric Mann–Whitney *U*‐test. The KOOS‐12 subscales were not treated as independent endpoints but as complementary domains within a single validated instrument, analysed to provide domain‐level detail rather than as separate hypothesis tests. For EQ‐5D‐3L and KOOS‐12 outcomes, mean differences with 95% confidence intervals (CIs) were calculated to indicate the magnitude and precision of between‐group differences. For proportions, CIs were also calculated when comparing groups.

To address baseline disparities in KOOS‐12 and EQ‐5D‐3L scores between the ACLROA patients and controls, a responder analysis was performed, which determined the proportion of patients whose postoperative change exceeded the minimal important change (MIC) for each PROM. Previously published thresholds were adopted: 11.5, 13.7 and 5.5 points for the KOOS‐12 pain, function, and quality of life subscales, respectively, and 14.9 points for the total KOOS‐12 score [10]. For the EQ‐5D‐3L utility index, an MIC of 0.105 was applied. MIC analysis was not performed for the EQ‐VAS due to the lack of a well‐established MIC value in the literature [29].

Each PROM was aimed to measure a distinct construct as an independent outcome rather than to perform multiple hypothesis testing, except for HRQoL measured with the EQ‐5D‐3L and EQ‐VAS. For this reason, *p*‐values were not corrected when comparing satisfaction and pain Likert scales or KOOS‐12 scores. The *p*‐values for the EQ‐5D‐3L index and EQ‐VAS were adjusted using the Benjamini–Hochberg method [15]. A two‐sided *p*‐value < 0.05 was considered statistically significant.

## RESULTS

### Satisfaction

At the 1‐year follow‐up, 998 ACLROA patients and 1996 matched controls were included. The distribution of satisfaction levels differed statistically between groups (*p *= 0.016), with both groups reporting similarly high satisfaction rates—approximately four out of five patients were ‘satisfied’ or ‘very satisfied’ with their TKA outcome (Table 2). Despite this statistical difference, the absolute variation in satisfaction between groups was minimal, indicating comparable overall satisfaction following surgery.

**Table 2:** Results—Satisfaction cohort 1 year postoperatively.

	ACLROA		Controls	
	Number (*%*)	95% CI	Number (*%*)	95% CI
1. Very dissatisfied	34 (3.3)	2.2−4.4	53 (2.7)	2.0–3.5
2. Dissatisfied	51 (5)	3.7–6.5	146 (7.3)	6.1–8.5
3. Neither nor	97 (9.7)	7.9–12	186 (9)	8.0–11
4. Satisfied	284 (29)	26–31	641 (32)	30–34
5. Very satisfied	532 (53)	50–56	970 (49)	46–51
Total	998 (100)		1996 (100)	

*Note*: Chi‐square test (*p* = 0.016).

Abbreviations: ACLROA, group of patients treated with TKA following prior ACLR; Controls, control group of patients with osteoarthritis undergoing TKA with no prior major surgery to the operated knee, such as osteotomy, osteosynthesis or anterior cruciate ligament reconstruction; CI, confidence intervals of proportions.

### Pain

In the matched cohort of 441 ACLROA patients and 882 controls, preoperative pain was slightly less severe among ACLROA patients (*p* = 0.001). One year after surgery, this difference was no longer statistically significant (*p* = 0.263), and both groups reported comparable postoperative pain relief, with severe pain being uncommon (Table 3).

**Table 3:** Pain Likert scale results.

	Preoperatively				One year postoperatively			
	ACLROA		Controls		ACLROA		Controls	
	Number (*%*)	95% CI	Number (*%*)	95% CI	Number (*%*)	95% CI	Number (*%*)	95% CI
1. None	3 (0.7)	0.0–1.4	1 (0.1)	0.0–0.3	157 (36)	31–40	273 (31)	28–34
2. Very mild	8 (1.8)	0.6–3.0	4 (0.4)	0.0–0.8	140 (32)	27–36	277 (31)	28–35
3. Mild	30 (6.5)	4.5–9.1	35 (3.5)	2.7–5.3	72 (16)	13–20	184 (21)	18–24
4. Moderate	212 (48)	43–53	400 (46)	42–49	56 (13)	9.6–16	112 (13)	11–15
5. Severe	188 (43)	38–47	442 (50)	47–53	16 (3.6)	1.9–5.3	36 (4.1)	2.8–5.4
Total	441 (100)		882 (100)		441 (100)		882 (100)	

*Note*: Chi‐square tests: Preoperatively (*p* = 0.001), 1 year postoperatively (*p* = 0.263).

Abbreviations: ACLROA, group of patients treated with total knee arthroplasty following prior anterior cruciate ligament reconstruction; Controls, control group of patients with osteoarthritis undergoing total knee arthroplasty with no prior major surgery to the operated knee, such as osteotomy, osteosynthesis or anterior cruciate ligament reconstruction; CI, confidence intervals of proportions.

### KOOS‐12

Among 595 ACLROA patients and 1190 controls, preoperative KOOS‐12 scores were marginally higher in the ACLROA group. At 1 year, total KOOS‐12 scores remained slightly higher in this group, but the mean difference was small and below established thresholds for clinical importance. The proportion of patients achieving a MIC was high and similar between groups, indicating no clinically relevant difference (Table 4).

**Table 4:** Results of KOOS‐12 subscales and total KOOS‐12 scores.

Pre and postoperative KOOS‐12 scores						
KOOS‐12 scores	ACLROA		Controls		Mean difference (95% CI)	*p* value^a^
	Mean (SD)		Mean (SD)			
Preoperative						
Pain subscale	33 (12)		29 (12)		4 (2.6–5.4)	<0.001
Function subscale	33 (13)		28 (13)		5 (3.5–6.5)	<0.001
Quality of life subscale	17 (11)		17 (11)		0 (–1.3 to 1.3)	0.819
Total score	27 (10)		24 (10)		3 (1.9–4.1)	<0.001
One year postoperative						
Pain subscale	66 (16)		65 (16)		1 (–0.19 to 2.9)	0.148
Function subscale	61 (16)		58 (17)		3 (1.4–4.6)	0.001
Quality of life subscale	52 (19)		50 (19)		2 (–0.1 to 4.1)	0.083
Total score	59 (16)		58 (16)		1 (–0.6 to 2.6)	0.22

Achievement of MIC threshold in KOOS‐12 scores						
KOOS‐12 scores	ACLROA (*n* = 595)		Controls (*n* = 1190)		% Difference (95% CI)	*p*‐Value^b^
	≥MIC (%)	95% CI	≥MIC (%)	95% CI		
Pain subscale	519 (87)	84–90	1052 (88)	87–90	–1 (–4.3 to 2.3)	0.471
Function subscale	489 (82)	79–85	990 (83)	81–85	–1 (–4.8 to 2.8)	0.594
Quality of life subscale	539 (91)	88–93	1071 (90)	88–92	1 (–1.9 to 3.9)	0.694
Total score	506 (85)	82–88	1038 (87)	85–89	–2 (–5.5 to 1.5)	0.203

Abbreviations: ACLROA, group of patients treated with total knee arthroplasty following prior anterior cruciate ligament reconstruction; CI, confidence intervals; Controls, control group of patients with osteoarthritis undergoing total knee arthroplasty with no prior major surgery to the operated knee, such as osteotomy, osteosynthesis or anterior cruciate ligament reconstruction; KOOS‐12, a 12‐item short form of the Knee injury and Osteoarthritis Outcome Score; MIC, minimal important change at the individual level; SD, standard deviation.

^a^ Mann–Whitney *U*‐test,

^b^ Chi‐square test.

### EQ‐5D‐3L

The matched cohort included 551 ACLROA patients and 1102 controls. Both groups demonstrated substantial improvement in HRQoL from baseline to 1 year after TKA. Postoperative EQ‐5D‐3L index and EQ‐VAS scores were slightly higher in the ACLROA group, indicating comparable or marginally better perceived health status (Table 5). Although these between‐group differences reached statistical significance, their magnitude remained below established thresholds for clinical relevance. In the responder analysis, 46% of ACLROA patients and 51% of controls exceeded the MIC threshold for the EQ‐5D‐3L index (*p* = 0.047), corresponding to a small absolute difference of approximately 5%, suggesting equivalent clinically relevant improvement of HRQoL in both groups.

**Table 5:** Results of EQ‐5D‐3L index and EQVAS health scores.

Pre and postoperative EQ‐5D‐3L scores						
	ACLROA		Controls		Mean difference (95% CI)	*p* value^a^
	Mean (SD)		Mean (SD)			
Preoperative						
EQ‐5D‐3L index	0.77 (0.11)		0.75 (0.11)		0.02 (0.009–0.031)	**0.008** b
EQVAS health	65.8 (23)		63.5 (22)		2.3 (0.3–4.3)	**0.028**
1 year postoperative						
EQ‐5D‐3L index	0.89 (0.11)		0.88 (0.11)		0.01 (0.001–0.021)	**0.038** b
EQVAS health	78.6 (18)		76.8 (19)		1.8 (0.0–3.8)	**0.036**

Achievement of MIC thresholds in EQ‐5D‐3L scores						
EQ‐5D‐3L	ACLROA (*n* = 551)		Controls (*n* = 1102)		% Difference (95% CI)	*p*‐Value^c^
	≥MIC (*%*)	95% CI	≥MIC (*%*)	95% CI		
EQ‐5D‐3L index	253 (46)	42–50	563 (51)	48–54	−5 (−10 to 0)	**0.047**

*Note*: Bold values indicate statistically significant *p*‐values (*p* < 0.05).

Abbreviations: ACLROA, group of patients treated with total knee arthroplasty following prior anterior cruciate ligament reconstruction; CI, confidence intervals; Controls, group of patients with osteoarthritis undergoing total knee arthroplasty with no prior major surgery to the operated knee, such as osteotomy, osteosynthesis or anterior cruciate ligament reconstruction; EQ‐5D‐3L, the 3‐level version of EQ‐5D is a standardised measure of health‐related quality of life developed by the EuroQol Group; MIC, minimal important change at the individual level; PROM, patient‐reported outcome measure; SD, standard deviation.

^a^ Mann–Whitney *U*‐test.

^b^ Adjusted using the Benjamini–Hochberg procedure controlling the false discovery rate across multiple PROM comparisons.

^c^ Chi‐square test.

## DISCUSSION

The most important finding of the present study was that patients with prior ACLR achieved 1‐year PROs after TKA that were comparable to those of matched controls without previous knee surgery.

A detailed evaluation of individual PROM domains demonstrated that while some intergroup differences reached statistical significance, namely the KOOS function subscale, total KOOS‐12 score, EQ‐5D‐3L, and EQ‐VAS, their magnitude remained below established minimal clinically important differences [7, 24]. These variations are therefore unlikely to represent clinically meaningful disparities between groups and likely reflect slightly higher baseline scores in the ACLROA group rather than a differential treatment effect. MIC analyses supported this interpretation, with high and comparable responder proportions for all KOOS‐12 subscales and total score (Table 4), while the EQ‐5D‐3L demonstrated only a 5% lower responder rate among ACLROA patients (Table 5).

Several mechanisms may help explain the slightly higher subjective outcomes observed in the ACLROA group. First, preoperative scores were already higher in this group, which may reflect a lower preoperative symptom burden at the time of TKA. Second, although the groups were matched by age at the time of TKA, patients with a history of ACL injury typically sustain their initial injury much earlier in life and tend to remain physically active thereafter. This long‐term activity profile may help preserve neuromuscular capacity and physiological reserve, potentially facilitating functional recovery following TKA [14]. Third, prior experience of ACL surgery may influence patient expectations and lead to a more realistic appraisal of surgical outcomes, positively influencing satisfaction [17, 19]. While these explanations are consistent with existing literature, they should be regarded as hypotheses rather than definitive causal mechanisms, as the present registry‐based study lacked variables to directly test these relationships.

Overall, the study′s results indicate that a history of ACLR does not adversely affect PROs after TKA. This is noteworthy because previous studies have reported that TKA in ACL‐reconstructed knees can be technically more demanding, often associated with higher complication rates, longer operative times and an increased need for revision‐style components and intraoperative soft‐tissue releases [5, 28]. In the present study, implant type was included in the matching process, ensuring that such differences did not influence the comparative analysis of PROs. However, the study did investigate whether these complexities affect longer‐term PROs. Comparable observations have been made in a recent matched‐cohort analysis by Jones et al., which found lower rates of aseptic revision and periprosthetic joint infection at 2 years, as well as fewer early complications such as manipulation under anaesthesia, wound disruption and medical events within 90 days in the ACLR‐TKA group compared with matched controls [16]. Likewise, in a systematic review, Chaudhry et al. pooled five retrospective case‐control studies (318 ACLR; 455 controls; mean follow‐up ≈3.4 years) and found no differences in postoperative knee pain, function and satisfaction after TKA between patients with and without prior ACLR, despite longer operative times and signals for higher reoperation in the ACLR cohorts [7]. Nevertheless, this review was limited by small samples and heterogeneity [7]. The present study confirms the absence of inferior PROs in ACLROA patients in a large, registry‐based matched study that utilised domain‐specific PROM analyses at 1 year.

## LIMITATIONS

This registry‐based, matched‐cohort study has several limitations. First, a nationwide, uniform PROM programme was not established until 2021, and a high number of TKA cases lacking PROMs data had to be excluded (Supporting Information: Table S2). The generalisability of the findings may be limited by transfer bias. Excluded ACLROA patients (Supporting Information: Table S2) were overall demographically similar to those included across all PROM cohorts (Table 1), but a greater proportion had undergone TKA before 2016, which reflects the late implementation of the PROM program. This difference is not expected to have influenced the results, as the same temporal pattern of TKA was observed among the excluded controls.

Second, PROM heterogeneity across clinics prevented analysis of all instruments in the same cohort, resulting in four separate PROM subsets. Matching was performed within each subset to reduce bias, but inter‐clinic variation in PROM collection may still have influenced results.

Third, PROM formats changed during the study period, with pain and satisfaction reporting shifting from VAS to five‐point Likert scales after 2021. The SAR provided standardised, converted data, ensuring comparability between groups. Similarly, only EQ‐5D‐3L data were analysed to avoid heterogeneity introduced by the later adoption of the EQ‐5D‐5L.

Fourth, the observation horizon was set to 1 year, which limits inferences about longer‐term PROs after TKA. While this timeframe provides valid insight into early subjective recovery, it does not allow for assessment of prosthesis survival, as most revisions typically occur beyond the first postoperative year. Nonetheless, a recent systematic review and meta‐analysis indicated that PROs obtained beyond 12–24 months typically change little and are often undermined by attrition and missing data, reducing their interpretability. Hence, a 1‐year assessment is still likely to yield clinically meaningful information [23].

Fifth, registry accuracy may be affected by unregistered prior ACLRs in the SAR, given that the SKLR was not established until 2005. The completeness of the SAR could not be verified for ACLRs performed before 2005, which may have led to an underestimation of the true number of ACLROA cases.

Finally, residual confounding from unmeasured factors cannot be excluded. Variables such as activity level, psychosocial variables, surgeon experience, and surgical volume may have influenced the findings. As TKA in ACLROA patients is generally regarded as technically more demanding than TKA in the standard knee OA population, it is likely that ACLROA procedures were performed by a smaller group of high‐volume, more experienced surgeons. In contrast, the control cases may have been treated by surgeons with a broader range of experience and caseloads. Matching for these parameters was not feasible, as such data are unavailable in the registry.

## CONCLUSION

In this study, prior ACLR did not adversely affect PROs 1 year after TKA. Satisfaction, pain relief, knee function and HRQoL were comparable between patients undergoing TKA after prior ACLR and those without prior knee surgery. Despite the presence of statistically significant differences in some PROM domains, the magnitudes were small and lacked clinical relevance. These findings indicate that a history of ACLR should not be considered a negative prognostic factor when counselling patients before TKA. Surgeons can reassure such patients that comparable outcomes are achievable, helping to set realistic expectations and guide treatment planning in everyday clinical practice.

## AUTHOR CONTRIBUTIONS

Ioannis Syrikas contributed to the study design, to the application for the necessary ethical approvals, acquisition and statistical analysis of data, drafted and wrote the manuscript, approved the final version, and is accountable for the integrity of the work. Ioannis Karikis contributed to the study design, to the interpretation and statistical analysis of data, critically revised the manuscript for important intellectual content, approved the final version for publication and is accountable for ensuring the accuracy and integrity of all aspects of the work. Neel Desai contributed to the study design, to the interpretation of data, critically revised the manuscript for important intellectual content, approved the final version for publication, and is accountable for ensuring the accuracy and integrity of all aspects of the work. Georgios Tsikandylakis contributed to the study design, application for the necessary ethical approvals, acquisition, and statistical analysis of data, critically revised the manuscript for important intellectual content, approved the final version for publication, and is accountable for ensuring the accuracy and integrity of all aspects of the work.

## CONFLICT OF INTEREST STATEMENT

The authors declare no conflicts of interest.

## ETHICS STATEMENT

The study received approval from the Swedish National Ethical Review Authority (Etikprövningsnämnden: Basic application, Ref No: 2021‐03780; Amendment application, Ref No: 2022‐03180‐02). Data extraction was approved by the Centre of Registers Västra Götaland (SAR) and by Region Stockholm (SKLR). All data were pseudonymized prior to extraction and analysis to ensure confidentiality. The Centre of Registers Västra Götaland handled the pseudonymization process and retained the key code securely for up to 1 year after data extraction, allowing patient identification if required.

## Supporting information

Supplementary tables_20251128.

## Data Availability

Data sharing is restricted by the Ethical Review Authority and data protection regulations. To access the data, a separate application must be submitted and approved by the relevant authorities.
