# Supplementary material for: Patients with previous anterior cruciate ligament reconstruction achieve equally good outcomes after total knee arthroplasty: A Swedish registry‐based study of 7755 patients
Source: Knee Surg Sports Traumatol Arthrosc. 2025 Dec 23;34(9):3377–86. doi: 10.1002/ksa.70240 (PMC13502416; doi:10.1002/ksa.70240)
Supplement: Supplementary file 1 [file KSA-34-3377-s001.docx]

| **Supplementary Table S1. Demographic characteristics of unmatched PROMs cohorts** | | | | | | | | |
| --- | --- | --- | --- | --- | --- | --- | --- | --- |
| **Factor** | **Satisfaction Likert scale** | | **Pain Likert scale** | | **KOOS-12** | | **EQ-5D-3L** | |
|  | **ACLROA** | **Controls** | **ACLROA** | **Controls** | **ACLROA** | **Controls** | **ACLROA** | **Controls** |
| **Number of cases** | 1088 | 46402 | 502 | 21576 | 671 | 29587 | 631 | 29717 |
| **Age**  Mean (SD) | 60 (8.3) | 70 (8.5) | 60.1 (8) | 69.8 (8.5) | 59.7 (8.2) | 69.8 (8.4) | 59.9 (8.1) | 69.9 (8.4) |
| **BMI**  Mean (SD) | 28.2 (3.8) | 28.7 (4.3) | 28.1 (3.8) | 28.6 (4.3) | 28.3 (3.8) | 28.8 (4.3) | 28.2 (3.8) | 28.7 (4.3) |
| **Sex** n (%) |  |  |  |  |  |  |  |  |
| Male | 729 (67) | 19556 (42) | 325 (65) | 8846 (41) | 442 (66) | 12666 (43) | 417 (66) | 12434 (42) |
| Female | 359 (33) | 26846 (58) | 177 (35.3) | 12730 (59) | 229 (34) | 16921 (57) | 214 (34) | 17283 (58) |
| **Side** n (*%*) |  |  |  |  |  |  |  |  |
| Right | 559 (51) | 24089 (52) | 407 (81) | 18701 (87) | 341 (51) | 15425 (52) | 317 (50) | 15492 (52) |
| Left | 529 (49) | 22313 (48) | 95 (19) | 2875 (13) | 330 (49) | 14162 (48) | 314 (50) | 14225 (48) |
| **ASA score**  n (*%*) |  |  |  |  |  |  |  |  |
| 1 | 371 (34) | 7505 (17) | 161 (32) | 3628 (18) | 234 (35) | 5217 (18) | 218 (35) | 5052 (18) |
| 2 | 627 (58) | 30054 (68) | 304 (61) | 14211 (69) | 388 (58) | 19056 (67) | 362 (58) | 18937 (68) |
| 3 | 88 (8.1) | 6728 (15) | 36 (7.2) | 2844 (14) | 48 (7.2) | 4061 (14) | 50 (7.9) | 3953 (14) |
| 4 | 0 (0) | 43 (0.1) | 0 (0) | 15 (0.1) | 0 (0) | 22 (0.1) | 0 (0) | 19 (0.1) |
| 5 | 0 (0) | 1 (0) | 0 (0) | 0 (0) | 0 (0) | 0 (0) | 0 (0) | 0 (0) |
| **Type of articulation**  n (*%*) |  |  |  |  |  |  |  |  |
| CR | 996 (91.6) | 43697 (94.4) | 459 (91.6) | 20403 (94.8) | 622 (92.8) | 28053 (95) | 572 (90.8) | 28264 (95.3) |
| PS | 80 (7.4) | 2047 (4.4) | 33 (6.6) | 863 (4) | 41 (6.1) | 1123 (3.8) | 48 (7.6) | 1062 (3.6) |
| CCK | 9 (0.8) | 419 (0.9) | 7 (1.4) | 213 (1) | 6 (0.9) | 308 (1) | 8 (1.3) | 277 (0.9) |
| Hinged | 2 (0.2) | 113 (0.2) | 2 (0.4) | 39 (0.2) | 1 (0.1) | 33 (0.1) | 2 (0.3) | 54 (0.2) |
| **Year of TKA** n (*%*) |  |  |  |  |  |  |  |  |
| 2000 – 2015 | 209 (19) | 13895 (30) | 94 (19) | 6321 (29) | 168 (25) | 10850 (37) | 189 (30) | 12219 (41) |
| 2016 – 2021 | 879 (81) | 32507 (70) | 408 (81) | 15255 (71) | 503 (75) | 18737 (63) | 442 (70) | 17498 (59) |
| *Mean (standard deviation), Absolute (relative frequencies)*  *BMI,* body mass index*; ASA score,* American Society of Anaesthesiologists Classification*; ACLROA, group of patients treated with total knee arthroplasty following prior* anterior cruciate ligament reconstruction*; Controls*, control group of patients with osteoarthritis undergoing *total knee arthroplasty* with no prior major surgery to the operated knee, such as osteotomy, osteosynthesis or anterior cruciate ligament reconstruction*; TKA,* total knee arthroplasty; *KOOS-12*, a 12-item short form of the Knee injury and Osteoarthritis Outcome Score; *EQ-5D-3L*, the 3-level version of EQ-5D is a standardised measure of health-related quality of life developed by the EuroQol Group; *CR*, cruciate retaining; *PS*, posterior stabilized; *CCK*, constrained condylar knee; | | | | | | | | |

| **Supplementary Table S2. Demographic characteristics of patients excluded due to missing PROM data** | | | | | | | | |
| --- | --- | --- | --- | --- | --- | --- | --- | --- |
| **Factor** | **Satisfaction Likert scale** | | **Pain Likert scale** | | **KOOS-12** | | **EQ-5D-3L** | |
|  | **ACLROA** | **Controls** | **ACLROA** | **Controls** | **ACLROA** | **Controls** | **ACLROA** | **Controls** |
| **Number of excluded cases** (%) | 2366 (69) | 173921 (79) | 2952 (86) | 198747 (90) | 2783 (81) | 190736 (87) | 2823 (82) | 190606 (87) |
| **Age** Mean (SD) | 58.6 (8.7) | 69.34 (8.9) | 58.9 (8.7) | 69.5 (8.8) | 58.9 (8.7) | 69.5 (8.8) | 58.9 (8.7) | 69.5 (8.8) |
| **BMI** Mean (SD) | 28.3 (4) | 29.2 (4.6) | 28.3 (4) | 29.1 (4.5) | 28.2 (4) | 29.1 (4.6) | 28.2 (4) | 29.1 (4.6) |
| **Sex** n (%) |  |  |  |  |  |  |  |  |
| Male | 1578 (67) | 71165 (41) | 1982 (67) | 81875 (41) | 1865 (67) | 78055 (41) | 1890 (67) | 78287 (41) |
| Female | 788 (33) | 102756 (59) | 970 (33) | 116872 (59) | 918 (33) | 112681 (59) | 933 (33) | 112319 (59) |
| **Side** n (*%*) |  |  |  |  |  |  |  |  |
| Right | 1233 (52) | 91288 (53) | 1385 (47) | 96676 (49) | 1451 (52) | 99952 (52) | 1475 (52) | 99885 (52) |
| Left | 1133 (48) | 82633 (47) | 1567 (53) | 102071 (51) | 1332 (48) | 90784 (48) | 1348 (48) | 90721 (48) |
| **ASA score** n (*%*) |  |  |  |  |  |  |  |  |
| 1 | 895 (38) | 18038 (17) | 1105 (38) | 21915 (17) | 1032 (37) | 20326 (17) | 1048 (37) | 20491 (17) |
| 2 | 1277 (54) | 69112 (65) | 1600 (54) | 84955 (66) | 1516 (55) | 80110 (66) | 1542 (55) | 80229 (66) |
| 3 | 187 (7.9) | 18485 (17.5) | 239 (8.1) | 22369 (17.3) | 227 (8.2) | 21152 (17.4) | 225 (8) | 21260 (17.4) |
| 4 | 2 (0.1) | 225 (0.2) | 2 (0.1) | 253 (0.2) | 2 (0.1) | 246 (0.2) | 2 (0.1) | 249 (0.2) |
| 5 | 0 (0) | 8 (0) | 0 (0) | 9 (0) | 0 (0) | 9 (0) | 0 (0) | 9 (0) |
| **Type of articulation**  n (*%*) |  |  |  |  |  |  |  |  |
| CR | 1998 (85.1) | 162162 (93.7) | 2535 (86.4) | 185456 (93.7) | 2372 (85.8) | 177806 (93.7) | 2422 (86.3) | 177595 (93.6) |
| PS | 332 (14.1) | 9855 (5.7) | 379 (12.9) | 11039 (5.6) | 371 (13.4) | 10779 (5.7) | 364 (13) | 10840 (5.7) |
| CCK | 11 (0.5) | 563 (0.3) | 13 (0.4) | 769 (0.4) | 14 (0.5) | 674 (0.4) | 12 (0.4) | 705 (0.4) |
| Hinged | 8 (0.3) | 504 (0.3) | 8 (0.3) | 578 (0.3) | 9 (0.3) | 584 (0.3) | 8 (0.3) | 563 (0.3) |
| **Year of TKA** n (*%*) |  |  |  |  |  |  |  |  |
| 2000 – 2015 | 1219 (52) | 134845 (78) | 1334 (45) | 142419 (72) | 1260 (45) | 137890 (72) | 1239 (44) | 136521 (72) |
| 2016 – 2021 | 1147 (48) | 39076 (22) | 1618 (55) | 56328 (28) | 1523 (55) | 52846 (28) | 1584 (56) | 54085 (28) |
| *Mean (standard deviation), Absolute (relative frequencies)*  *BMI,* body mass index*; ASA score,* American Society of Anaesthesiologists Classification*; ACLROA, group of patients treated with total knee arthroplasty following prior* anterior cruciate ligament reconstruction*; Controls*, control group of patients with osteoarthritis undergoing *total knee arthroplasty* with no prior major surgery to the operated knee, such as osteotomy, osteosynthesis or anterior cruciate ligament reconstruction*; TKA,* total knee arthroplasty; *KOOS-12*, a 12-item short form of the Knee injury and Osteoarthritis Outcome Score; *EQ-5D-3L*, the 3-level version of EQ-5D is a standardised measure of health-related quality of life developed by the EuroQol Group; *CR*, cruciate retaining; *PS*, posterior stabilized; *CCK*, constrained condylar knee. | | | | | | | | |
